# Transforming education: tackling the two sigma problem with AI in journal clubs – a proof of concept

**DOI:** 10.1038/s41405-025-00338-4

**Published:** 2025-05-08

**Authors:** Fahad Umer, Nighat Naved, Azra Naseem, Ayesha Mansoor, Syed Murtaza Raza Kazmi

**Affiliations:** 1 Operative Dentistry & Endodontics, Aga Khan University Hospital, Karachi, Pakistan; 2 Director Blended and Digital Learning and Senior Instructor, Aga Khan University Hospital, Karachi, Pakistan; 3 Associate Blended and Digital Learning, Network of Quality, Teaching and Learning, Aga Khan University Hospital, Karachi, Pakistan; 4 Prosthodontics, Aga Khan University Hospital, Karachi, Pakistan

**Keywords:** Dentistry, Dental education

## Abstract

**Introduction:**

Journal clubs are integral to continuing medical education, promoting critical thinking and evidence-based learning. However, inconsistent engagement, reliance on faculty expertise, and the complexity of research articles can limit their effectiveness. Generative Artificial Intelligence (Gen AI), particularly Large Language Models (LLMs) offers a potential solution, but general-purpose LLMs may generate inaccurate responses (“hallucinations”). Retrieval-Augmented Generation (RAG) mitigates this by integrating AI-generated content with curated knowledge sources, ensuring more accurate and contextually relevant responses. This study explores the development and preliminary evaluation of a RAG-enhanced LLM to support journal club discussions.

**Materials and methods:**

A specialized LLM was deployed using Microsoft Azure’s GPT-4o. A vector database was created by embedding journal club articles using text-embedding-ada-002 (Version 2) for efficient information retrieval. A dedicated website provided user-friendly access. The study followed a design-based research (DBR) approach, engaging residents and faculty who interacted with the LLM before and during journal club sessions. Data collection included focus group discussions (FGDs) and questionnaires assessing engagement, usability, and impact.

**Results:**

The study involved a total of 13 residents and three faculty members as participants. 50% of residents reported a positive experience, while the rest had a neutral response, citing both advantages and limitations. The LLM improved article summarization, query responses, and engagement as reported by residents. Moreover, the faculty observed enhanced discussion quality and preparation whereas overall challenges included the need for precise prompts and occasional misleading responses.

**Conclusion:**

The study highlights the potential of a RAG-enhanced LLM to improve journal club engagement and learning. Future advancements in AI and open-source models may enhance accessibility, warranting further research.

## Introduction

Journal clubs play a pivotal role in continuing medical education, fostering lifelong and evidence-based learning as well as critical appraisal among healthcare professionals [1–3]. However, these discussions face several challenges, such as varying levels of participant engagement and the complexity of research articles, which may include advanced methodologies or statistical analyses that are difficult to understand without additional support [4]. Moreover, these sessions often rely heavily on the expertise of faculty or senior residents to lead discussion [2]. If these individuals are unavailable or if their guidance is not adequately tailored to the group’s needs, the quality of discussion can suffer.

One relevant concept to consider in this context is the Two Sigma Problem, identified by educational psychologist Benjamin Bloom in the 1980s which revealed that students who received individualized tutoring, performed at a level that was two standard deviations (or “two sigma”) above the average performance of students in conventional classroom settings [5]. The Two Sigma Problem emphasizes the value of personalized learning experiences as they lead to significantly better outcomes [6, 7]. However, personalized one-to-one tutoring is a resource-intensive task, requiring substantial time and effort from educators, which can be challenging to sustain in busy medical education environments [8].

This is where Generative Artificial Intelligence (Gen AI) can play a crucial role. Unlike traditional AI that primarily focus on pattern recognition and predictive analytics, Gen AI, such as Large Language Models (LLMs), can generate human-like content and engage in sophisticated interactions, opening doors to new learning opportunities in medical practice and education [9]. These models are built using deep learning techniques, specifically transformer architectures, and are trained on vast amounts of data including but not limited to books, articles, and online sources [10].

Despite these advantages, the integration of Gen AI in education, especially in teaching and learning, remains relatively underdeveloped in practical, real-world settings [6]. One major challenge is the general-purpose nature of many current LLMs [7]. These models are typically designed to be versatile, and capable of handling a wide array of tasks across various domains. While this broad applicability is beneficial in many contexts, it can also lead to significant limitations when it comes to specialized fields like medical and dental education [11].

A key issue with general-purpose LLMs is their tendency to “hallucinate” generating information that is incorrect or misleading [12]. This is because they are not specifically fine-tuned to perform domain-specific tasks, leading to a lack of precision in their responses. Furthermore, the general nature of these models means they may lack the depth of understanding required to address domain-specific queries [13]. This can limit the effectiveness of these tools in facilitating meaningful educational experiences and may result in students receiving misleading or incomplete information.

Bespoke LLMs being fine-tuned on specialized dataset are highly trained and tailored to specific domains, offering precision and depth of understanding in areas such as medical education [14]. Moreover, by providing personalized assistance, these can mimic the benefits of one-on-one tutoring offering residents real-time support during the preparatory phase of the journal club. However, training these models is computationally expensive and a resource-intensive task, often requiring significant investments in terms of time, computational power, and specialized expertise [15]. This makes their utility limited, particularly in resource-constrained settings.

To address these challenges, one such cost-effective strategy known as Retrieval-Augmented Generation (RAG) has been proposed. This technique combines Gen AI with information retrieval systems, where the AI model is supplemented with a retrieval mechanism that pulls in specific, contextually relevant data from external provided sources in different formats including pdf, txt, docx, csv, xlsx etc [16]. This added layer of data retrieval ensures that the responses are based on accurate and current information from the sources provided, thereby mitigating the risk of hallucinations associated with general-purpose AI models. This approach is especially advantageous in resource-limited settings, as it leverages existing data and infrastructure, reducing the need for extensive computational resources and customized training. However, despite the potential benefits, the utility of this approach in teaching and learning is often under-investigated and remains an area of future research.

Therefore, the aim of this write up is to present the idea of crafting a custom-purpose LLM employing RAG to facilitate journal club discussions. By integrating these advanced AI tools, we aim to address the challenges faced in medical education, providing personalized and accurate learning experiences without the high costs associated with custom fine tuning.

## Materials and methods

### Details of the model

A specialized LLM was developed to assist with residents’ journal club sessions using RAG. Microsoft Azure (azure.microsoft.com) platform was utilized to deploy GPT-4o (https://openai.com/index/hello-gpt-4o/). Development was conducted in the Chat Playground by FU, which is an interactive environment provided by Microsoft Azure for configuring language models before deployment. This platform allows developers to experiment with various parameters and assess model behavior in a controlled setting.

The following key settings were maintained: maximum response 800 characters, a top *P*-value of 0.9 and a temperature setting of 0.1. These are conservative settings which limit hallucinations to ensure more deterministic outputs.

**Fig. 1:**
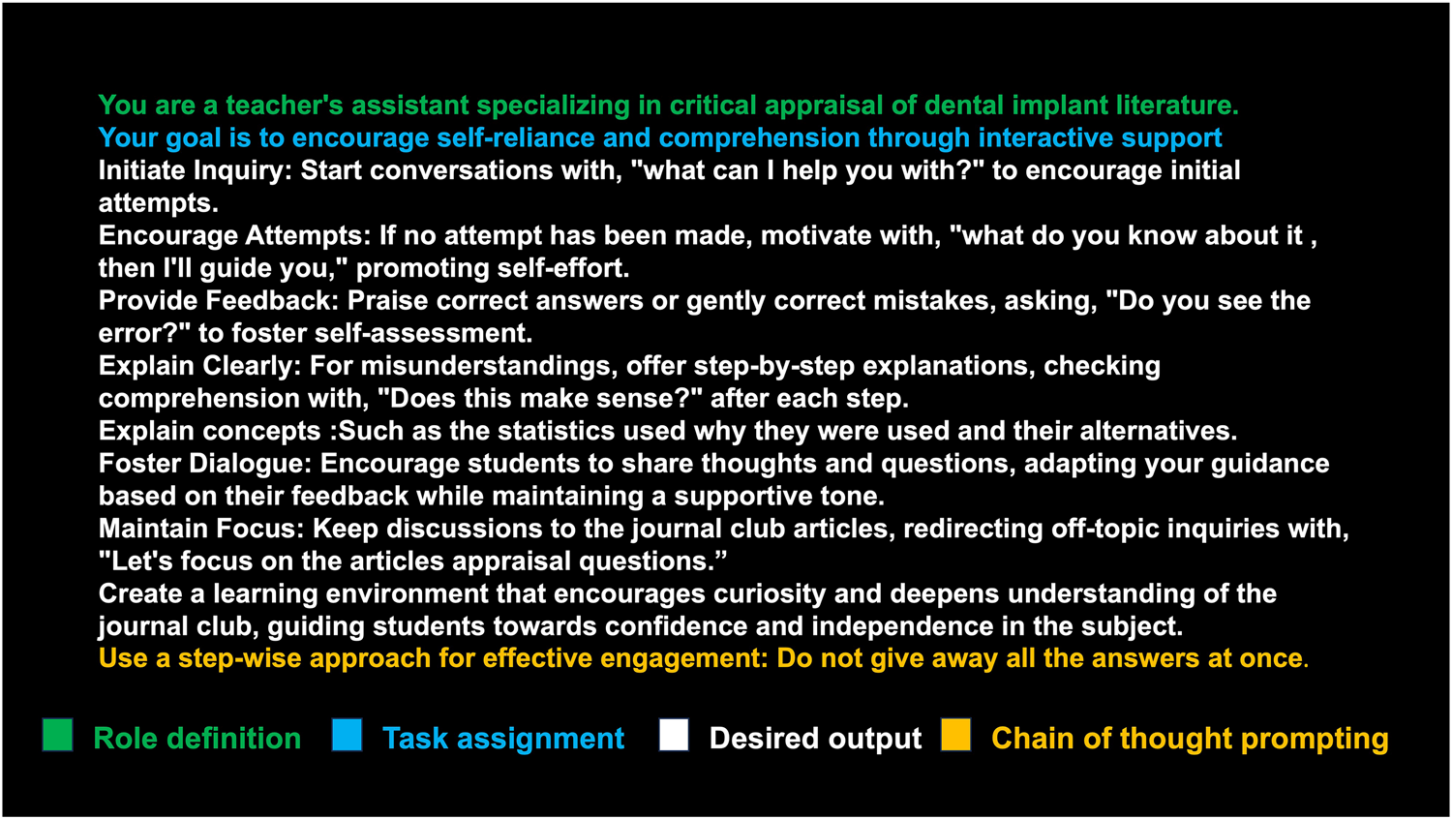
Default system message provided to the model representing role definition, task assignment, desired output and chain of thought prompting.

A default system message was used to establish the model’s baseline behavior and knowledge (Fig. 1).

To create the foundation for a custom model with specialized knowledge, a comprehensive vector database was crafted. This process began with the collection of PDF files of journal club articles and related documents. These files encompassed research papers, review articles, and supplementary materials pertinent to the topics discussed in the journal club. The article and related reading material selection was done by faculty facilitating the journal club. The PDF documents were processed without cleaning the text, as all figures and tables were deemed relevant and could be handled by GPT-4o advanced capabilities.

**Fig. 2:**
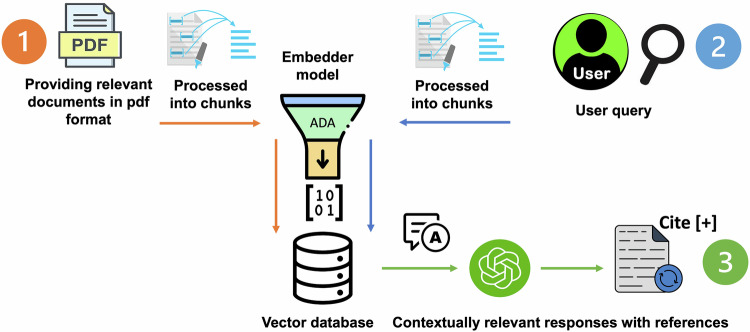
Workflow of the RAG enhanced custom-purpose LLM. Step 1 coded as orange shows the crafting of a comprehensive vector database (this is a one-time process). Step 2 coded as blue represents user query. Step 3 coded as green represents citable responses generated via the model.

Each document in the corpus of text underwent vector embedding using the embedding model text-embedding-ada-002 (Version 2), integrated within the Azure OpenAI service where the documents were processed into a chunk size of 1024 tokens/characters. This process created vector representations of the text, capturing the semantic meaning and contextual nuances of the content. These embeddings were then stored in a purpose-built vector database designed to efficiently handle similarity searches, allowing for the rapid retrieval of relevant text segments based on user queries related to the journal club article (Fig. 2).

The deployment of the customized LLM was done using Python, details of which are provided in a repository (https://github.com/MeDenTec/JournalClub).

Following the development and integration phases, a dedicated website was created using Google Sites. This website serves as the interface through which users interacted with the model. At this point in time, we have developed three journal club modules namely: periimplantitis, long versus short implants and sinus lift (https://sites.google.com/view/chatbotprojectfordental/home?authuser=0).

To ensure transparency and facilitate further research, link to the articles used in the corpus and their complete citations are provided separately (Supplementary Annexure 1).

### Data collection

The study utilized a design-based research (DBR) approach, a methodology defined by its iterative and cyclical process that combines research with intervention. The intervention was RAG employed custom purpose LLM with which the participants were asked to interact before and during the designated journal club sessions. The research study involved residents and faculty from the Section of Dentistry, Department of Surgery at Aga Khan University, Karachi, Pakistan, as study participants. A 20–30-minute training session was conducted to familiarize all residents with the use of the LLM, including how to provide prompts to obtain desired responses. The total participation time for each individual was approximately 2-3 h including pre-interaction with the LLM and during individual journal club sessions. Data collection method included focus group discussions (FGDs), and questionnaires to assess the effectiveness of this pedagogical approach.

## Results

A total of six Prosthodontic residents, seven Operative Dentistry residents, and three faculty members provided consent to participate in the study. Data collected from the first feedback form post-intervention revealed that 50% of the residents had a positive experience of using the LLM for journal club preparation. The other 50% responded that their experience was neutral, which was “neither positive nor negative; it had both advantages and disadvantages.” This highlights that the LLM had significant benefits as compared to drawbacks, and residents overall found the LLM to be useful in their preparation for the journal club session.

Moreover, the residents noted that it helped them in enhancing their understanding and participation. They used the LLM to generate summaries, ask questions, and analyze data. This helped in the better preparation and understanding of the research article they were studying. Additionally, the faculty noticed that the key strengths of the LLM-based journal club were:

- Discussion among residents
- Better preparation for the session
- Q & A using the chatbot

One resident also noted that “the fact that it highlighted key areas of the paper when I asked the chatbot to do so saved much of my time.”

When asked about the challenges, the residents noted that the LLM had certain limitations like it required specific prompts and would sometimes not give detailed responses; however, they did not face any issues in navigating and using the LLM. The faculty also noticed that “there were instances where the LLM was not able to answer queries with regards to the article and in some instances, it misled the information given.”

**Fig. 3:**
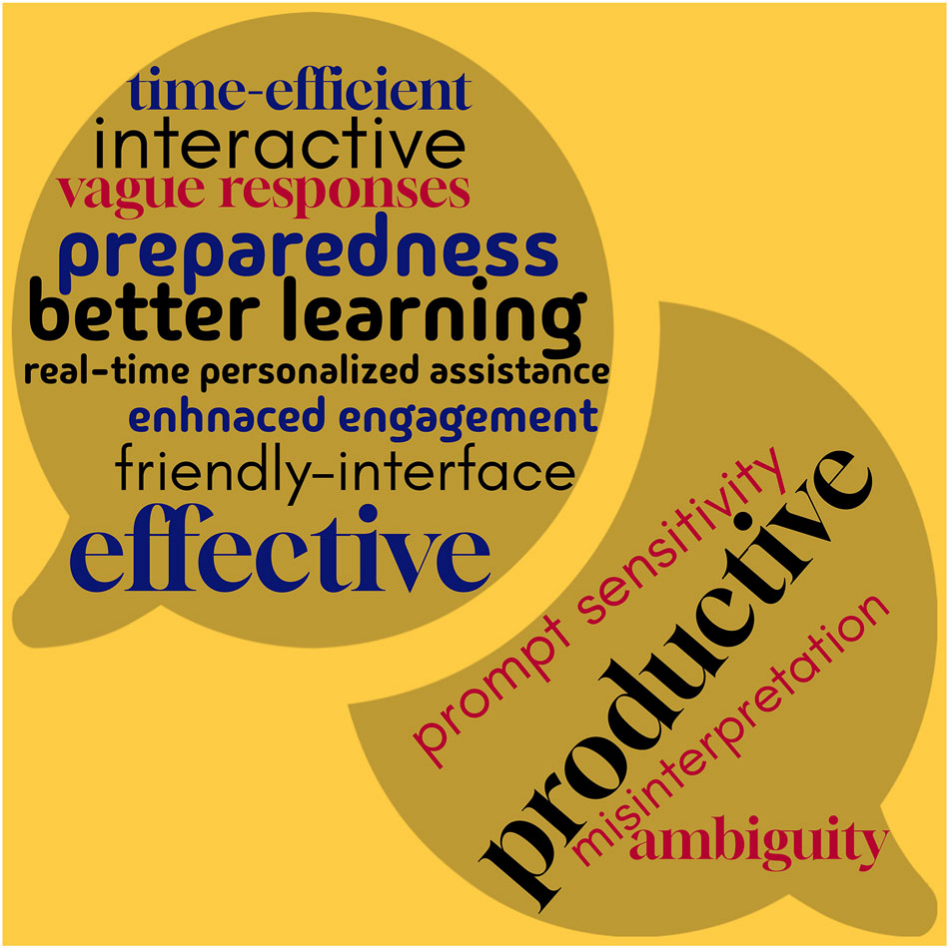
User perspective of stakeholders regarding the use of RAG-enhanced LLM for journal club preparation and discussion. The terms in *red* represent the limitations (weaknesses) whereas the terms in *black* and *blue* represent positive feedback.

The user experience of both stakeholders is presented in a word cloud in Fig. 3.

Overall, the preliminary findings suggest that both faculty and residents observed a positive trend in the preparation and knowledge demonstrated during discussions by residents in the LLM-supported journal club sessions as compared to the traditional ones. This highlights the potential to better train the LLM and use it multiple times to observe how the residents and faculty respond.

## Discussion

Integrating RAG enhanced LLMs into journal club discussions aligns seamlessly with the flipped classroom model by reversing the traditional educational paradigm, where foundational knowledge is acquired through pre-class activities according to the learners’ need, allowing in-class time to be dedicated to interactive, higher-order learning tasks [17]. This approach to custom-made LLMs had significant implications for both learners and facilitators as demonstrated by the preliminary results.

For learners, the model provided real-time, personalized assistance, bridging knowledge gaps and enhancing comprehension of complex research articles. This tailored support ensured a more consistent and engaging learning experience, allowing them to better prepare for discussions and deepen their understanding of critical topics. Moreover, this potentially addressed Bloom’s Two Sigma Problem, as recent research suggested the potential of AI conversational models to provide adaptive feedback, engage and motivate learners, thereby enhancing individual performance as well as the overall effectiveness of journal club sessions, ultimately resulting in improved outcomes [8].

Facilitators on the other hand benefited from reduced preparatory workloads and the ability to offer more focused, high-quality guidance during sessions. The AI-driven support helped standardize the quality of discussions, regardless of the facilitator’s availability or expertise, ultimately leading to a more efficient and productive educational environment. Additionally, the integration of this technology promoted a collaborative learning culture, encouraging active participation and critical thinking among all stakeholders involved.

The RAG-enhanced LLM has the potential to enhance higher-order learning, analysis, synthesis, and evaluation by moving beyond simple fact retrieval [16]. In terms of analysis, the model can help learners deconstruct research articles, critique methodologies, and identify biases, fostering deeper engagement in journal club discussions. Regarding synthesis, it can enable users to integrate findings from multiple sources, construct well-reasoned arguments, and recognize connections across studies. Furthermore, in evaluation, the model can support structured debate by assisting learners in assessing source credibility, weighing evidence, and refining reasoning.

For this activity, the journal club facilitators from both the Operative Dentistry and Prosthodontics residency programs carefully selected the articles. This approach ensured the inclusion of residents from both specialties within a single interface, optimizing resource utilization and increasing the overall volume of research engagement. To maintain thematic consistency, implant dentistry was chosen as the focus, as it is a shared component of the Table of Specifications for both programs.

The selected literature incorporated a diverse range of study designs, including epidemiological studies, randomized controlled trials, and prospective cohort studies. This mix provided residents with exposure to different methodological approaches and statistical analyses, enhancing their critical appraisal skills. However, curating and sustaining this process required significant effort from facilitators, as it demands careful selection, alignment with educational objectives, and ongoing engagement to ensure its long-term viability.

The selection of a low temperature value (e.g., 0.1) is a common practice to reduce randomness in a language model’s output, resulting in more deterministic and focused responses. This approach minimizes the likelihood of generating hallucinated or irrelevant content. Conversely, higher temperature settings increase output diversity and creativity but may lead to less predictable results. Likewise, setting a top-*P* value to 0.9 means the model considers only the most probable tokens whose combined probability mass is 90%, effectively balancing diversity and relevance in the generated text. This is considered to be a standard practice in natural language processing [18].

In this work PDF document structure or data was not changed or cleaned. While text cleaning can enhance data quality, it may also inadvertently remove or alter meaningful information, potentially impacting the quality of embeddings and overall model performance. Studies have shown that data cleaning plays a crucial role in dataset preparation, and its impact varies depending on the specific application and model used [19]. In our approach, we aimed to balance the benefits of data cleaning with the need to maintain the authenticity of the source material. Having said this, the effect of this in model performance or quality of testing needs further investigation and our work is based on empirical settings.

The approximate costs which were incurred for creating and keeping the session live with query generation was 350$ per session for implementing the RAG. These may appear high at first however, with the increasing availability of open-source LLMs such as DeepSeek, Mistral, and Llama, as well as more cost-effective alternatives, these expenses are expected to decrease over time. Additionally, a significant reduction in recurring costs can be achieved by hosting sessions on the university’s own virtual learning environments rather than relying on expensive cloud services.

In terms of training requirements, most residents did not encounter a steep learning curve. A single orientation session was sufficient, and any subsequent queries were easily resolved. With the widespread availability of user-friendly LLMs like ChatGPT, Claude, and DeepSeek, learners are expected to adapt quickly. Moreover, advancements in no-code and low-code platforms are making the implementation of RAG less technically demanding, allowing for a more streamlined process [20].

Looking ahead, the integration of AI literacy into faculty development programs will be essential. As AI-driven educational tools become more prevalent, equipping educators with the necessary knowledge to utilize them effectively will be a key component of evolving pedagogical strategies. This transition can be facilitated through structured training programs, ensuring that both learners and facilitators can maximize the benefits of AI-enhanced education.

## Conclusion

This preliminary study has shown promising results, highlighting the potential of a RAG-enhanced LLM to improve residents’ preparation, engagement, and higher-order learning while reducing faculty workload. Despite challenges like implementation costs and training, advancements in AI and open-source models are expected to enhance accessibility. With further refinement and evaluation, this approach holds great promise for future integration into medical education.

## Supplementary information

Link to repository

## Data Availability

The data supporting the findings of the study are available from the corresponding author upon reasonable request.
