# Supplementary material for: Transforming education: tackling the two sigma problem with AI in journal clubs – a proof of concept
Source: BDJ Open. 2025 May 8;11:46. doi: 10.1038/s41405-025-00338-4 (PMC12062218; doi:10.1038/s41405-025-00338-4)
Supplement: Supplementary file 1 — Link to repository [file 41405_2025_338_MOESM1_ESM.docx]

**Session 1: Periimplantitis**

**Further Reading:**

1. Romandini (2023)

<https://drive.google.com/file/d/1HYrfQuXtqc7SlA9Hni0UCG94Z0N0l-5m/view?usp=drive_link>

1. Monje (2023) <https://drive.google.com/file/d/1YVKW5eTDQg0q7HvY_4UFJBIZ6kR0IABd/view?usp=drive_link>
2. Smeets (2014) <https://drive.google.com/file/d/1lEignFP_PLNxGS9j9QC4H3i2IgbNuBKh/view?usp=drive_link>
3. Derks (2016) <https://drive.google.com/file/d/1m_NVI1JpOQZ2TMl_8zJY8yya3DCCx0mW/view?usp=drive_link>
4. Derks (2015)

<https://drive.google.com/file/d/1mXeg5TbBSBzwe1STyYbwpGBRyowxqZd/view?usp=drive_link>

1. Seroni (2024)

<https://drive.google.com/file/d/1hdGiR5_mNi_y7q5pJO3g3yif-2yqJE07/view?usp=drive_link>

1. Anitua (2021)

<https://drive.google.com/file/d/1T4TPyfWbR_6eE-60yeYq3_L4XiwlQdLo/view?usp=drive_link>

1. Ruiz‐Romero

<https://drive.google.com/file/d/1HTkZxHrQV4ln-2GEbjNsqNiw-6Bp7EiI/view?usp=drive_link>

**Session 2: Short versus Long Implants**

**Further Reading**

1. Bitinas (2021) <https://drive.google.com/file/d/1wuDZLlEddbGm8ZAYFCbHLlxeP4_f1Fi4/view?usp=sharing>
2. Thoma (2024) <https://drive.google.com/file/d/1OC5dwoArpK3o1gfl9ezWhH5jVIGjCKzB/view?usp=sharing>
3. Al-Hashedi (2016)

<https://drive.google.com/file/d/1qiiaAPqKRGb01maeVBBej-GxMFg6IUxh/view?usp=sharing>

1. Liang (2024) <https://drive.google.com/file/d/1C2WN8uImptpdWJTp2PrFcMiMxISuvgqj/view?usp=sharing>
2. Jain (2016)

<https://drive.google.com/file/d/1M8ECR2D-F0kRRyiyHR7ffkLaYh6lKojv/view?usp=sharing>

1. John (2018)

<https://drive.google.com/file/d/1ZQHhr_OwkerdTBkfY5jUdmo2gAIu6-8P/view?usp=sharing>

1. Mittal (2019) <https://drive.google.com/file/d/12Ru7l6GIo_1g7PvqtSZrEylSPl_7j4f6/view?usp=sharing>
2. Palacios (2018)

<https://drive.google.com/file/d/1EoTr0csTypVUDBsvJ8EdD7TFI-TTbk3Z/view?usp=sharing>

1. Papaspyridakos (2018) <https://drive.google.com/file/d/1UBc_XxX6iKfxFRkCQDAQywRbkGirxr3M/view?usp=sharing>
2. Rameh (2020)

<https://drive.google.com/file/d/18LRmrjUiSyMLFGGV1LBA7f0SOyKVTWT/view?usp=sharing>

1. Rossi (2015)

<https://drive.google.com/file/d/1-u2U0lo0lYTJrpzQJ3-rsIzZaI9JjtSA/view?usp=sharing>

1. Thoma (2021) <https://drive.google.com/file/d/11T6J4bOOiRXyneC2jXDJyDPLOnbG10I7/view?usp=sharing>
2. BDJ part 3

<https://drive.google.com/file/d/1dkkpEd72bfYn-eTgnVM_KRhLNchmgu5W/view?usp=sharing>

1. BDJ part 4 <https://drive.google.com/file/d/1LET84jEl9gh8SDv98SAmxauj13_H7u48/view?usp=sharing>

**Session 3: Sinus Lift**

**Further Reading**

1. Jamcoski-2023

<https://drive.google.com/file/d/1RPXLO_efMGwhyQLwHMkn8NqAwlYLIDp/view?usp=sharing>

1. Al-Dajani-2016

<https://drive.google.com/file/d/1cZ0MlVQjAarjwk9E9v8cg9waYegvUGt/view?usp=sharing>

1. Bacevic-2021

<https://drive.google.com/file/d/1ms_WL1TivXqeSmmrPb5XADFK1KhoLUC/view?usp=sharing>

1. Corbella-2015

<https://drive.google.com/file/d/1QJ4_hmLvu0LlxXtAdNoCBQlIediumx1/view?usp=sharing>

1. Gandhi-2024

<https://drive.google.com/file/d/113aLLl2FCeUf3w92SODMUqUqPB2qcyv/view?usp=drive_link>

1. Gandhi-2021

<https://drive.google.com/file/d/14XiPbhlCs7DB_96SpwS_VBmWNKuIEy1/view?usp=sharing>

1. Raghoebar-2019

<https://drive.google.com/file/d/1nWce8N5QrFL6qUeA9GiT3Wpc-oi2Q-GQ/view?usp=sharing>

1. Kumar-2019

<https://drive.google.com/file/d/1c4t98KQYdwqvbrkRGJn8NcCF8gOm92E8/view?usp=sharing>

1. Molina-2022

<https://drive.google.com/file/d/12VAKsbx2va2Cv5DJcZWZihQd1ZPg2PvG/view?usp=sharing>

1. Rosano-2010

<https://drive.google.com/file/d/1ED1Qp-Kx8ThIksFJkzewJ6H0yhhR1esv/view?usp=sharing>

1. Jain-2015(stats)

<https://drive.google.com/file/d/1nMJAPp5Crja4sbciMsLVYHycZ24MfkX/view?usp=sharing>
